# Triple Test Plus Rapid Cognitive Screening Test: A Combination of Clinical Signs and A Tool for Cognitive Assessment in Older Adults

**DOI:** 10.3390/diagnostics9030097

**Published:** 2019-08-15

**Authors:** Saadet Koc Okudur, Ozge Dokuzlar, Derya Kaya, Pinar Soysal, Ahmet Turan Isik

**Affiliations:** 1 Department of Geriatric Medicine, Manisa State Hospital, Manisa 45040, Turkey; 2 Unit for Brain Aging and Dementia, Department of Geriatric Medicine, Faculty of Medicine, Dokuz Eylul University, Izmir 35340, Turkey; 3 Department of Geriatric Medicine, Faculty of Medicine, Bezmialem Vakif University, Istanbul 34093, Turkey

**Keywords:** cognitive screening instruments, dementia, mild cognitive impairment, cognitive impairment, Rapid Cognitive Screen, Triple Test

## Abstract

Less time-consuming, easy-to-apply and more reliable cognitive screening tests are essential for use in primary care. The aim of this study was to investigate the diagnostic value of the Turkish version of the Rapid Cognitive Screen (RCS-T) and Triple Test individually and the combination of RCS-T with each sign and Triple Test to screen elderly patients for cognitive impairment (CI). A total of 357 outpatients aged 60 or older, who underwent comprehensive geriatric assessment, were included in the study. Presence or absence of attended alone sign (AAS), head-turning sign, and applause sign was investigated. The mean age of the patients was 74.29 ± 7.46. Of those, 61 patients (28 men, 33 women) had Alzheimer’s disease (AD), 59 patients had mild cognitive impairment (MCI) (29 men, 30 women), and 237 (80 men, 157 women) were cognitively robust. The sensitivity of the combination of RCS-T and negative for AAS for CI, AD and MCI is 0.79, 0.86 and 0.61, respectively; the specificity was 0.92, 0.93 and 0.92, respectively; and the positive and negative predictive values revealed good diagnostic accuracy. The combination of RCS-T and negative for AAS is a simple, effective and rapid way to identify possible CI in older adults.

## 1. Introduction

Memory complaints, which should be distinguished from cognitive impairments (CI) related to neurodegenerative diseases such as Alzheimer’s disease (AD), are quite prevalent among older adults [1,2]. It is difficult to devote time for detailed neuropsychological tests during a busy clinical day, which is the first approach to determine whether or not a patient requires further clinical evaluation for CI [3]. Therefore, less time-consuming, easy-to-apply and more reliable cognitive screening tests (which are essential to accurately diagnose patients with CI, especially for primary care settings) may help primary care professionals determine whether it is necessary to refer a patient to a tertiary care or a memory center for cognitive diseases [4].

Elaborate medical history, physical examination, and neurocognitive assessment should be performed to identify the etiology of memory loss [5]. The Rapid Cognitive Screen (RCS) is a brief screening tool derived from the Saint Louis University Mental Status (SLUMS) examination for the detection of cognitive dysfunction in primary care settings [6] and the Turkish version of the RCS (RCS-T) is validated in Turkish older adults [7]. Additionally, inclusion of some clinical signs of neurodegenerative diseases leading to dementia, such as the attended alone sign (AAS) [8], head-turning sign (HTS) [9,10] and applause sign (AS) [11] in cognitive assessment, may play a significant role in the diagnostic process [4,12].

The Triple Test consisting of AAS, HTS and AS has been shown to be a simple, rapid and effective screening tool for detecting cognitive impairment, and deterioration of daily living activities in older adults [4,12]. AS is also called the clapping test, three-clap test, or signe d’applause. The evaluation is as follows: The patient is asked to clap three times as quickly as possible after the examiner had performed a demonstration. When the patient claps three times, this is considered negative for AS. When the patient claps more than three times, this is considered positive for AS. Patients clapping less than three times are considered pathologic, and positive for AS, as reported previously [13,14]. In the literature [9,15], HTS is assessed while the patient’s caregiver sat silently at a 45° angle, approximately 1 m behind the patient, who is encouraged to speak about their cognitive function history. When the patient turns her/his head away from the collocutor and towards the caregiver(s) for help, the patient is considered positive for HTS. When the patient did not want any help, this is considered negative for HTS. AAS is evaluated based on whether the patient attended the clinic with a family member, caregiver, or friend. When the patient is accompanied, this is considered negative for attended alone sign [15].

Early definitive diagnosis of cognitive impairment may be difficult in busy clinic settings, in which the main purpose of combining tests is to ensure early recognition of patients with cognitive impairment and referral to a higher center.

Nevertheless, it is not known whether the combination of the RCS-T test, a clinical screening tool, and the Triple Test, a test based on clinical symptoms, has positive effects on the management of memory problems. Therefore, in the present study we aimed to investigate the diagnostic value of the RCS-T and Triple Test individually and the combination of RCS-T with each sign and Triple Test to screen elderly patients for CI.

## 2. Materials and Methods

A total of 357 patients aged over 60 years, who were admitted to the geriatric outpatient clinic with memory complaints between June 2014 and February 2019, and underwent comprehensive geriatric assessment, were included in the study. All the participants, who provided informed consent to participate in the study, were evaluated by comprehensive geriatric assessment, including physical and mental status examination and laboratory evaluation. The ethics committee of Dokuz Eylul University, Turkey, approved the study protocol with decision number 2018/22-19, and the project identification code is 4241-GOA (September 13, 2018). Each participant or a legal guardian provided written, informed consent to participate in the study. We carried out this study in accordance with the provisions of the Declaration of Helsinki.

Major and minor CI was diagnosed according to the Diagnostic and Statistical Manual of Mental Disorders (fifth edition) diagnostic criteria [16] and AD was diagnosed according to National Institute on Aging–Alzheimer’s Association workgroup’s criteria [17]. The cognitively robust group consisted of patients who had memory complaints despite normal cognitive assessments and those who attended our geriatric outpatient clinic for prevention programs because of other medical issues.

Socio-demographic characteristics of the patients, including gender, age and education, were recorded. All patients were examined to see if they had hearing loss, cataracts, hypertension, diabetes mellitus, coronary artery disease, peripheral vascular disease, hyperlipidemia, cerebrovascular disease, congestive heart failure, depression, dementia, or polypharmacy in their medical history. For each patient, a comprehensive geriatric assessment, including detailed and complete physical and mental examinations, was performed [18]. Patients were evaluated based on the RCS-T [7], Mini-Mental State Examination (MMSE) [19], Geriatric Depression Scale [20], Instrumental Activities of Daily Living [21], Basic Activities of Daily Living Scale [22], Clinical Dementia Rating, and a clock drawing test. For the patients with CI, further evaluations, such as laboratory examination and brain imaging (essential for the differential diagnosis CI) were also performed.

The signs were assessed by two physicians (S.K.O and O.D.), who were blinded to the diagnosis of the patients. High interrater agreements were observed (1.00 for AAS, 0.98 for HTS, and 1.00 for AS). Presence or absence of AS, AAS, and HTS was investigated.

The RCS test includes 3 items: recall of 5 words, a clock drawing test, and the ability to remember a story [6]. Scores ranged from 0 and 10. The RCS-T optimal cut-off scores were ≤ 4 for dementia and ≤ 6 for mild cognitive impairment (MCI) [7].

Patients with unilateral hearing impairment or blindness and deafness, and those considered to be too sick to be questioned, including those in delirium, were excluded. Those who had been taking medications that might influence their thinking or memory, who had previous head trauma resulting in unconsciousness and/or a period of memory impairment, who were unable to provide informed consent to participate in the study, who cannot speak Turkish, or who were illiterate, were excluded. Reversible CI related to vitamin B12 or folic acid deficiency, hypothyroidism, subdural hematoma, hyponatremia, medications (e.g., corticosteroids, antipsychotics, and benzodiazepines), substance abuse problems and the institutionalized older adults were excluded.

The package program, Statistical Package for Social Sciences (SPSS) version 23.0 for Windows (SPSS Inc., Chicago, IL, USA), was used for the statistical analysis. Descriptive statistics are presented as means ± standard deviations or percentages. Nominal variables were evaluated by Pearson’s χ2-test. Kruskal–Wallis test was used to analyze the presence of non-normal distribution, and continuous variables with normal distribution were assessed with one-way ANOVA (shown as the P_1_ value in Table 1). Adjustment according to age and gender was carried out by multinomial logistic regression analysis (shown as the P_2_ and P_3_ value in Table 1). Sensitivity refers to proportion of correct positive classifications, and specificity refers to proportion of correct negative classifications. *p*-value < 0.05 was considered significant. Sensitivity, specificity, and positive and negative predictive values (PPV, NPV) were calculated for diagnosis of any CI, AD, and MCI. A sample size of 214 participants was calculated as the minimum required size to be within a 5% of the true proportion.

## 3. Results

A total of 357 outpatients (137 men, 220 women) with a mean age of 74.29 ± 7.46 years were assessed. Of the patients, 61 (28 men, 33 women) were diagnosed with AD, 59 were diagnosed with MCI (29 men, 30 women) and 237 (80 men, 157 women) were cognitively robust (controls). There was a significant difference among AD, MCI and controls in terms of gender and age; there were more female participants in controls (*p* < 0.05) and older patients in AD group (*p* < 0.001). There was no difference in the patients with MCI, AD, and controls with regards to total years of education or educational level of the participants (for each, *p* > 0.05). There was no difference for the number of drugs between the groups (*p* > 0.05). After comprehensive geriatric assessment, when MMSE, SLUMS, RCS-T, clinical dementia rating test, clock-drawing test, Geriatric Depression Scale, Basic Activities of Daily Living Scales and Instrumental Activities of Daily Living were evaluated, there was a significant difference between the groups (*p* < 0.001) except for Geriatric Depression Scale. Descriptive data of the patients are summarized in Table 1. After adjustments for age and gender by multinomial logistic regression analysis MMSE, RCS-T, SLUMS, clock-drawing test (CDT), Clinical Dementia Rating (CDR), Basic Activities of Daily Living (BADL), and Instrumental Activities of Daily Living (IADL) were still significant in the AD group compared to the control group (*p* < 0.001). When MCI and control group were compared, statistical significance was maintained for MMSE, SLUMS, RCS-T, CDR, CDT (*p* < 0.001); but BADL (*p* = 0.259) and IADL (*p* = 0.169) showed no statistical significance.

Patients’ neuropsychological profiles were assessed based on the presence or absence of AS, HTS and AAS and the neuropsychological tests (MMSE, SLUMS, RCS-T and clock-drawing test). The sensitivity and specificity, and PPV and NPV values of the tests are shown in Table 2.

## 4. Discussion

The present study demonstrated that RCS-T and the Triple Test are easy to perform together and are highly specific tools to screen elderly patients with CI, AD or MCI while the combination of RCS-T and negative for AAS is sensitive and specific enough to accurately screen those with CI.

Discrimination between cognitively normal and CI in older adults, especially in early stage, requires long, time-consuming cognitive tests and sophisticated evaluations. For this purpose, many tests are currently available for cognitive screening. Out of them, the sensitivity and specificity of MMSE, Montreal cognitive assessment, Turkish version of SLUMS, and Cognitive state test screening tests are 0.91 and 0.95; 0.81 and 0.78; 0.84 and 0.87; and 0.81 and 0.78, respectively [19,23,24,25]; however, they tend to be time consuming and may be inappropriate for both bedside clinical evaluation and daily clinical practice, especially in the primary care setting. In a recent study, SLUMS-derived RCS [6,7] has been reported to be a simple, fast and practical tool that is sensitive and specific enough to screen CI in older adults and reflects deterioration in daily living activities [7] which is confirmed once again in the present study.

In addition, it is highly important that physicians consider cognitive screening tools as well as the early clinical signs of specific neurodegenerative diseases, such as HTS, AAS, and AS in order to make a differential diagnosis. The neuropsychological properties of HTS are potentially heterogeneous, including amnesia, impaired comprehension and lack of insight [10]. Previous studies showed that AAS is effective in detecting cognitively robust patients [12,26]. AAS is also thought to give information about instrumental activities of daily living and noncognitive functions in the same domain such as mobility, gait, balance and vision [4,27]. AS is a motor perseveration indicative of frontal lobe dysfunction in different types of dementia [28]. In a recent study, the combination called the Triple Test has been demonstrated to be a simple, quick and efficient screening tool for detecting CI [4].

The present study showed that the combination of RCS-T or the Triple Test positivity has a good sensitivity for discriminating between patients with CI, AD, and MCI (0.86, 0.88 and 0.62, respectively) and also better with higher NPV for all (0.92, 0.97 and 0.93, respectively). It means that the combination can correctly identify older patients without CI among those with memory problems, and that it can be used for older adults with memory complaints in bedside evaluation or primary care setting. Hence, those who are negative for the combination may not need to be referred to the relevant memory centers for detailed cognitive assessment. Additionally, in the present study, RCS-T plus AAS has good sensitivity and high specificity to detect CI in older adults.

When the current results were compared with our previous ones [4], it was found that sensitivity and specificity of the Triple Test were not similar in these studies, which can be explained by gender and educational differences, considering the fact that some cultural and methodological differences should be kept in mind while assessing the results of the studies [29].

Of particular note in this study, the RCS-T test together with HTS showed a remarkable NPV (0.97), which may be a signal or alert to closely monitor individuals with the possibility of MCI or to refer to the memory centers. With this finding, using RCS-T and positive for HTS together can be considered as a daily questionnaire because the RCS-T test is a reliable and valid measure, and indeed doctors inevitably evaluate HTS during routine history taking. Unfortunately, there is no specific applicable measurement to differentiate those with MCI from those that are cognitively robust, but measurements are increasingly being investigated for early detection of AD/MCI. Recently, it has been shown that Addenbrooke’s Cognitive Examination III has a sensitivity of 0.77 and specificity 0.92 in capturing individuals with MCI in Japan [30], and a sensitivity of 0.63 and specificity of 0.63 in Malay [31]. Our finding, with a sensitivity of 0.78 and specificity of 0.87, is also compatible with the aforementioned studies.

RCS-T alone is also very valuable due to the fact that it differentiated cognitively robust older adults accurately from those who had MCI or cognitive impairment. In the present study, RCS-T is combined with Triple Test, which is performed by physicians during the physical examination. It is striking that specificity of the combination was high enough to show that individuals with a good test performance are healthy. Furthermore, patients who have poor performance for either RCS-T or Triple Test are more likely to be cognitively impaired, with a sensitivity of 0.86. Therefore, older people with memory problems could be better directed for further neurocognitive analysis if either of them is positive.

The strengths of this study are the large sample size and that all the cases included in the study were over 60 years of age. As far as we are concerned, this is the first study to evaluate the diagnostic value of the combination; however, it has still some limitations. Since it was conducted at a memory center, the generalizability of our findings might be limited. Another limitation is that HTS requires the presence of a person accompanying the patient to the clinic.

## 5. Conclusions

Our study has demonstrated that the combination of RCS-T and negative for AAS is simple, effective and rapid way to identify possible CI in older adults. The combination of RCS-T and Triple Test has the ability to identify the cognitively robust older adults with subjective memory complaints, while those with poor performance for either of them may require further neurocognitive evaluation for CI. Therefore, the combination of both tests could serve as an indicator of those patients in need of further neurocognitive analysis, especially in daily busy clinical practice.

## Figures and Tables

**Table 1:** Descriptive characteristics of participants (*n* = 357).

	Control (*n* = 237)	MCI (*n* = 59)	AD (*n* = 61)	P_1_	P_2_	P_3_
**Women (%)**	66.2	51.7	53.3	0.041	-	-
**Age**	72.45 ± 7.08	76.37 ± 6.09	78.95 ± 7.63	<0.001	-	-
**Education (year)**	8.61 ± 3.84	7.98 ± 3.76	8.48 ± 4.09	0.390	-	-
**Number of drugs**	5.33 ± 3.33	5.65 ± 3.02	5.92 ± 3.07	0.214	-	-
**MMSE**	28.26 ± 1.71	25.25 ± 3.44	17.92 ± 4.86	<0.001	<0.001	<0.001
**SLUMS**	25.14 ± 3.19	19.50 ± 4.56	10.88 ± 4.64	<0.001	<0.001	<0.001
**RCS-T**	8.28 ± 1.52	5.42 ± 2.13	2.15	<0.001	<0.001	<0.001
**CDR**	0.0 ± 0.1	0.5 ± 0.2	1.5 ± 0.6	<0.001	<0.001	<0.001
**CDT**	4.7 ± 0.8	4.2 ± 1.2	2.2 ± 1.3	<0.001	<0.001	<0.001
**GDS**	2.8 ± 3.3	3.1 ± 3.3	3.7 ± 3.3	0.139	-	-
**BADL**	95.46 ± 5.87	93.15 ± 13.22	83.58 ± 18.28	<0.001	<0.001	0.259
**IADL**	14.99 ± 3.13	13.58 ± 3.94	8.07 ± 4.95	<0.001	<0.001	0.169

AD, Alzheimer’s disease; BADL, Basic Activities of Daily Living; CDR, Clinical Dementia Rating; CDT, clock-drawing; GDS, Geriatric Depression Scale; IADL, Instrumental Activities of Daily Living; MCI, mild cognitive impairment; MMSE, Mini-Mental State Examination; P_1_, *p*-values for comparison of three groups; P_2_, *p*-values for comparison of AD and control group after adjusted for age and gender; P_3_, *p*-values for comparison of MCI and control group after adjusted for age and gender; RCS-T, Turkish version of the Rapid Cognitive Screen test; SLUMS, Saint Louis University Mental Status Examination. Score range for each of the assessments: BADL, 0 (worst)–100 (best); CDR, 0 (best)–3 (worst); CDT, 0 (worst)–5 (best); GDS, 0 (best)–15 (worst); IADL, 0 (worst)–17 (best); MMSE, 0 (worst)–30 (best), RCS-T, 0 (worst)–10 (best); SLUMS, 0 (worst)–30 (best). All data are expressed as means ± standard deviations.

**Table 2:** Assessment of the diagnostic value of the RCS-T and Triple Test individually and the combination of RCS-T with each sign and Triple Test.

	Each Test		Combınatıons of RCS + Each Sign			Combınatıons of RCS + Trıple Test	
	RCS-T	Triple	RCS-T and PHTS	RCS-T and PAS	RCS-T and NAAS	RCS-T and Triple	RCS-T or Triple
***CI***							
Sensitivity %	85.83	29.66	58.47	29.66	79.66	29.17	86.44
Specificity %	88.19	96.51	96.94	98.69	92.14	98.7	85.59
PPV %	78.63	81.40	90.79	92.11	83.93	92.11	75.56
NPV %	92.48	72.70	81.92	73.14	89.79	72.76	92.45
***AD***							
Sensitivity %	86.67	42.37	69.49	40.48	86.44	39.49	88.33
Specificity %	90.91	93.75	94.77	98.61	93.03	98.89	85.76
PPV %	65.82	58.14	73.21	85.71	71.83	86.82	56.38
NPV %	97.12	88.82	93.79	88.73	97.09	87.06	97.24
***MCI***							
Sensitivity %	73.33	15.52	78.79	16.95	61.02	15.25	61.54
Specificity %	88.19	96.51	87.06	98.69	92.14	99.13	87.11
PPV %	61.11	52.94	44.07	76.92	66.67	81.82	45.28
NPV %	92.89	81.85	96.94	82.18	90.17	81.95	92.89

AD, Alzheimer’s disease; CI, Cognitive impairment; MCI, Mild cognitive impairment; NAAS, Negative for attended alone sign; NPV, Negative predictive value; PAS, Positive for applause sign; PHTS, Positive for head-turning sign; PPV, Positive predictive value; RCS, Rapid Cognitive Screen test.
